# Clustering of Activity-Related Behaviors in Relation to Self-Reported Causes of Stress among Pre-Adolescents: Results from a National Epidemiological Study

**DOI:** 10.3390/life13030622

**Published:** 2023-02-23

**Authors:** Rena I. Kosti, Thomas Tsiampalis, Matina Kouvari, Ioannis Gketsios, Aikaterini Kanellopoulou, Venetia Notara, George Antonogeorgos, Andrea Paola Rojas-Gil, Ekaterina N. Kornilaki, Areti Lagiou, Demosthenes B. Panagiotakos

**Affiliations:** 1 Department of Nutrition and Dietetics, School of Physical Education, Sports and Dietetics, University of Thessaly, 42132 Trikala, Greece; 2 Department of Nutrition & Dietetics, School of Health Sciences & Education, Harokopio University, 17671 Athens, Greece; 3 Faculty of Health, University of Canberra, Canberra 2617, Australia; 4 Department of Public and Community Health, Laboratory of Hygiene and Epidemiology, School of Public Health, University of West Attica, 12243 Athens, Greece; 5 Department of Nursing, Faculty of Health Sciences, University of Peloponnese, 22100 Tripoli, Greece; 6 Department of Preschool Education, School of Education, University of Crete, 74100 Rethimno, Greece

**Keywords:** clustering, health promotion, physical activity, pre-adolescents, stress

## Abstract

An epidemiological study was conducted among 1728 10–12-year-old students (55.1% girls) and their parents during 2014–2016 in Greece. This study aimed to identify the dominant clusters of physical activity/sedentariness among preadolescents and investigate their association with self-reported sources of stress. Children’s physical activity levels and sources of stress were evaluated using validated questionnaires that assessed daily hours of activities, both on weekdays and on weekends, including physical activity, screen-based sedentary time, and non-screen-based sedentary time. The k-means algorithm of cluster analysis was applied. Three clusters of children’s physical activity/sedentariness were revealed. Cluster 1 was characterized as “Inactive-Non sedentary”, cluster 2 as “Active –Non-screen sedentary”, and cluster 3 as “Inactive-Sedentary”. Parental needs/expectations were associated with physical activity patterns (*p* = 0.009), i.e., children assigned to the third and second clusters had 36% and 51% lower odds to be stressed due to parental requirements [(OR for cluster 3 = 0.64, 95% CI = 0.41–0.99), (OR for cluster 2 = 0.49, 95% CI = 0.32–0.76)], compared with their first-cluster counterparts. Considering the need to promote physical activity in early life stages, the identification of these complex activity-related patterns along with their significant interaction with parental expectations as a cause of stress could enhance the effectiveness of targeted behavior change interventions among those parent–child dyads most in need.

## 1. Introduction

Since the beginning of this century, compelling evidence has underscored the beneficial effects of physical activity and the detrimental consequences of sedentariness on various health outcomes, well-being, quality of life, and longevity [1,2]. The pleiotropic benefits of physical activity on human health start from the early stages of life and continue across the lifespan. However, a mounting body of evidence underlines the declining trends in physical activity and increased sedentary behavior across childhood and adolescence [3,4]. The period that a child transitions into adolescence is a determinant of the formulation of various behaviors including habits related to physical activity [5]. Given that the adoption of healthy behaviors tracking into adulthood has a cumulative effect on health, the instilling of healthy habits during this period is of crucial importance [6,7].

In the past few years, findings from a systematic review and meta-analysis, which synthesized evidence from 163 observational studies, challenged the view that “physical activity” and “sedentary habits” are mutually exclusive behaviors, implying that engagement in high levels of sport is not necessarily displaced by excessive amounts of television watching or vice versa by children and adolescents [8]. Towards this concept, clusters of physical activity and sedentary behavior habits have been reported in the literature demonstrating that many different patterns exist [9,10]. The configuration of these patterns is the result of the combination of psychosocial, personal, and environmental factors [11]. In this context, findings from a systematic review suggested a reciprocal and bidirectional association between psychological stress and activity-related behaviors [12]. It has been also remarked that in youth, the higher the exposure to more stressors, the lower the likelihood to be physically active and the higher the likelihood to adopt a sedentary lifestyle [13]. Moreover, the literature suggests that causes of stress are dependent on the adolescents’ stage [14]. However, there is scarce evidence regarding the dominant stressors in preadolescence [15] and their association with activity-related patterns.

The hypothesis of the present study is that sources of stress have differential effects on activity-related patterns. Thus, the aim of the present study was to identify the dominant clusters of physical activity/sedentariness among preadolescents and to investigate their association with self-reported sources of stress.

## 2. Materials and Methods

### 2.1. Design and Setting

The present study is a school-based cross-sectional study, conducted in the school period of 2014–2016. Forty-seven primary schools were randomly selected among the school of the Peloponnese peninsula (i.e., Sparta, Kalamata, and Pyrgos), the capital city of the island of Crete (i.e., Heraklion), and the greater Athens metropolitan area, using a list provided by the Greek Ministry of Education and Religious Affairs. The sampling was representative of the reference population according to the census in 2011; the area covered has approximately 75% of the total Greek population and represents large urban and rural municipalities.

### 2.2. Sample

All children aged 10–12 years old attending the fifth and sixth grades of the selected primary schools, along with their parents, were invited to participate in the study. A total of 1728 children (54% girls) were finally enrolled; the participation rate within schools ranged from 95% to 100% without any significant differences between the studied areas.

### 2.3. Measurements

#### 2.3.1. Assessment of Other Children’s Characteristics

Sociodemographic characteristics, i.e., age, sex, place of birth, ethnicity, number of siblings, and birth order, were recorded during the interview. In addition, weight (in kg) and height (in m) were measured and recorded by a trained investigator using a scale and a measuring-tape, over skin-tight clothes with no shoes. Body mass index (BMI) was calculated (kg/m^2^), and children were then categorized as underweight, normal weight, overweight, or obese, according to the International Obesity Task Force BMI cut-off points [16]. Sleep duration (in hours) was calculated as the interval between the reported bedtime and the reported hour children usually woke up. Given that sleep duration was based on a self-reported bedtime and wake-up time, it was actually a self-reported sleep duration.

To evaluate children’s level of adherence to the Mediterranean diet, the KIDMED questionnaire was used, which was originally developed by Serra-Majem et al. (2004) [17] to combine the Mediterranean diet guidelines for adults, as well as the general dietary guidelines for children (i.e., breakfast skipping) in a single index. According to the KIDMED index, a score of 0–3 reflects poor adherence to the Mediterranean diet, a score of 4–7 describes average adherence with improvement needed to adjust intake to Mediterranean patterns, and a score of 8–12 is good adherence.

#### 2.3.2. Assessment of Children’s Physical Activity

Children’s leisure-time physical activity was estimated through a special, validated questionnaire, i.e., the Physical Activity and Lifestyle Questionnaire (PALQ) [18], in which children reported the mean daily hours, both on weekdays and weekends (0–1 h, 1–2 h, 2–3 h, 3–5 h, and >5 h), they spent (i) studying for school, (ii) computer use, (iii) TV watching, (iv) reading extracurricular books, (v) playing board games, and (vi) playing electronic games. In addition, children were asked if they were involved in a Sport Club, as well as the frequency (times per week (1 time, 2 times, 3 times, 4 times, 5 times, 6 times) and duration of their training each time (~30 min, 30–60 min, 60–90 min, >90 min)). Children were also asked about the time they walk on a daily basis for their various obligations (<15 min, 15–30 min, 31–45 min, 46–60 min, >60 min). Based on the aforementioned questions, three new variables were created, depicting the frequency (minutes/week) of (i) physical activity (e.g., playing sport, walking, etc.), (ii) screen-based sedentary time (e.g., frequency of TV watching, playing board games, etc.), and (iii) non-screen-based sedentary time (e.g., frequency of studying for school). Finally, children were asked about the method of travelling to school (walking, by car, another way (school bus, bicycle, etc.)), as well as the years of systematic physical exercise.

#### 2.3.3. Assessment of Sources of Stress in Children

Children’s sources of stress were evaluated using specific questions about the (i) parents’ requirements/expectations from them, (ii) teachers’ requirements/expectations from them (iii) school performance, (iv) pressed schedule of activities, and (v) peer’s pressure (i.e., from classmates or friends), based on the Adolescent Stress Questionnaire (ASQ) [19] translated and evaluated in several countries, including Greece [20].

#### 2.3.4. Assessment of Parents’ Physical Activity Level and Other Characteristics

Each parent provided information about their age (in years), ethnicity ((i) Greek, (ii) Other), place of birth, highest educational level ((i) Basic/Secondary level: Primary school/Graduate of high school/Graduate of general or technical lyceum, (ii) Higher: Graduate of Technical University/Graduate of Higher Education Institute/Postgraduate/PhD), occupational status ((i) Employed/Retired: State employee/Private employee/Freelance/Retired, (ii) Unemployed), marital status ((i) Married, (ii) Single: Widow-widower/Divorced/Cohabitation), as well as the annual family income. Parents’ physical activity was also recorded and evaluated according to weekly frequency and categorized as follows: (i) 0 times/week, (ii) 1–2 times/week, and (iii) more than 3 times/week. In addition, self-reported weight and height were recorded, and body mass index (BMI) was calculated (kg/m^2^); parents were then categorized as underweight, normal weight, overweight or obese, according to the WHO standards for the BMI cut-off points.

### 2.4. Statistical Analysis

#### 2.4.1. Cluster Analysis of Children’s Physical Activity Behaviors

The k-means algorithm of cluster analysis (CA) with the K-nearest-means classifier was applied to define the clusters of children with common physical activity behaviors. The frequency of physical activity, screen-based sedentary time, and non-screen-based sedentary time were converted into Z-scores (standardized) and entered into the cluster algorithm, which was run 100 times in order to reduce the effect of random splitting. The analyses were performed for two to five clusters, and the best cluster solution was chosen in terms of the amount of explained variation, the size (≥10% of the sample) and interpretation of each cluster, and its stability. Finally, a four-cluster solution was decided to be optimal.

#### 2.4.2. Other Data Analyses

Children’s and parents’ categorical characteristics are presented as relative frequencies (%) and continuous characteristics are presented as mean (Standard Deviation (SD)) values. Associations between the categorical characteristics and children’s clusters of physical activity behaviors were evaluated through the Pearson Chi-square test, while the One-way Analysis of Variance (ANOVA) was used for the continuous characteristics. The normality of continuous characteristics’ distribution was tested through the P-P plot and the Kolmogorov–Smirnov test. In addition, the association between the sources of stress and the children’s clusters of physical activity behaviors was evaluated through the Pearson Chi-square test, while logistic regression analysis was also performed in order to compare the different clusters of children regarding their odds to be stressed due to their parents’ requirements. Logistic regression results are presented as Odds Ratio (ORs) and their corresponding 95% Confidence Intervals (CIs). All statistical analyses were conducted using Stata 14.0 (M. Psarros & Assoc., Sparti, Greece).

## 3. Results

### 3.1. Children’s Physical Activity Patterns

**Figure 1:**
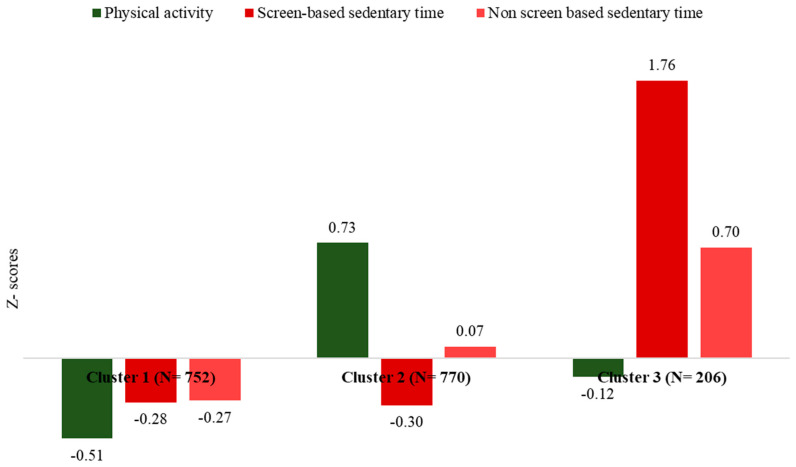
Physical activity patterns of children according to their physical activity time and sedentary time (screen-based and non-screen-based). Notes: The K−means algorithm of cluster analysis (CA) with the K−nearest-means classifier was applied to define the clusters of children with common physical activity behaviors. The frequency of physical activity, screen-based sedentary time, and non-screen-based sedentary time were converted to Z−scores (standardized) and entered into the cluster algorithm.

Several clustering schemes (i.e., with two, three, four, and five clusters) were applied, but the most informative was the one with three clusters (as revealed using the dendrogram and the Bayesian Information Criterion—BIC). The highest percentage of children belonged to Cluster 2, i.e., 45%, followed by Cluster 1, i.e., 43%, and Cluster 2, i.e., 12%. Figure 1 illustrates the results from the cluster analysis (presented as Z-scores) concerning children’s physical activity patterns, using the time engaged in physical activities, screen-based sedentary time, and non-screen-based sedentary time. In particular, cluster 1 was characterized by time spent on physical and sedentary activities (both screen-based and non-screen-based) below the average, and thus, named as “Inactive-Non-Sedentary pattern”. Cluster 2 was characterized by physical activity time above the average, but by screen-based sedentary time below the average and characterized as an “Active–Non-screen Sedentary pattern”, and cluster 3 was characterized by sedentary time above the average irrespective of screen-based, or non-screen based, and by physical activity time below the average and was thus characterized as an “Inactive-Sedentary pattern”.

### 3.2. Demographic, Lifestyle, and Activity-Related Characteristics of Children

In Table 1, the lifestyle characteristics of children according to the physical activity cluster to which they belonged are presented. Children with a more sedentary pattern reported unhealthier dietary habits, a higher likelihood to consume fast food, and fewer sleeping hours when compared to more physically active children. Significant differences were observed among the three clusters regarding all lifestyle and activity parameters, except for the frequency of breakfast consumption (*p* = 0.721) and the number of meals including snacks (*p* = 0.082). Regarding the other activity parameters, as presented, the highest screen-based and non-screen-based time was observed among the children belonging to the third cluster, while the highest physical activity time was observed among the children in the second cluster. In addition, children belonging to the second cluster were more likely to walk for their everyday obligations, while it is also worth noting the fact that children in the third cluster were more likely to consume and order more fast-food. Finally, the highest percentage of children with a high level of adherence to the Mediterranean diet was observed among children in the second cluster, while the lowest level of adherence to the Mediterranean diet was observed among children belonging to the first cluster.

In Table 2, the demographic and socioeconomic characteristics of the children and their parents, according to the physical activity cluster to which they belonged, are presented. In general, there were no significant differences among the physical activity clusters. However, it is worth noting the fact that the highest percentage (85%) of single-parent (mother) families was observed among the children belonging to the first cluster, while it is also worth noting the fact that the third cluster (sedentary pattern) was dominated by boys.

### 3.3. Prevalence of Stress and Its Main Sources According to Children’s Activity-Related Patterns

**Figure 2:**
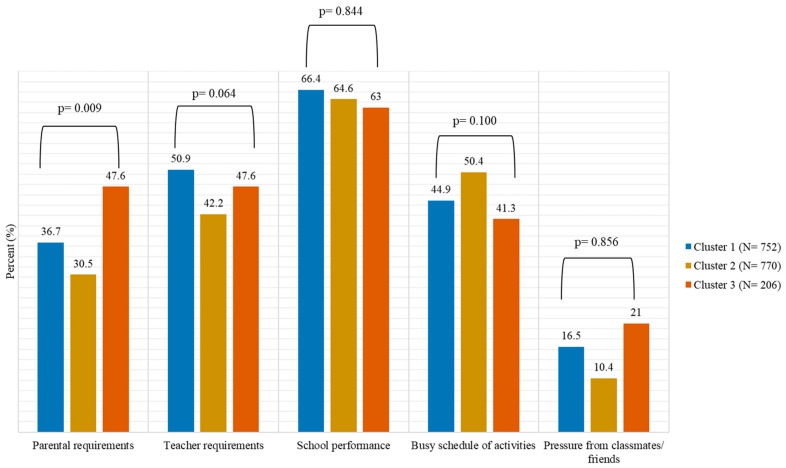
Sources of stress for children according to the cluster in which they belong. Notes: Cluster 1 was characterized by both physical activity time and sedentary time (both screen-based and non-screen-based), below the average; Cluster 2 was characterized by physical activity and non-screen-based sedentary time above the average, and by screen-based sedentary time below the average; Cluster 3 was characterized sedentary time (both screen-based and non-screen-based) above the average, and by physical activity below the average; *p*-value was based on the Pearson chi-square test.

Regarding the stress levels of children, it was found that 87.9% of the children reported having stress, yet there was no significant difference among the three different clusters in terms of activity-related behaviors (*p* = 0.135). However, after investigating the main sources of stress, parental requirements/expectations were found to be significantly associated with children's physical activity patterns (*p* = 0.009). Specifically, a higher percentage of children was found to be stressed due to their parental requirements in the third cluster (47.6%), followed by the children in the first cluster (36.7%). On the other side, the lowest percentage of children stressed due to their parents’ requirements was observed among the children in the second cluster (30.5%) (Figure 2). Compared to children belonging to the third cluster, those belonging to the first cluster had 36% lower odds to be stressed due to their parental requirements (OR = 0.64, 95% CI 0.41, 0.99), while those belonging to the second cluster had 51% lower odds to be stressed due to their parental requirements (OR 0.49, 95% CI0.32, 0.76).

## 4. Discussion

The present study aimed to explore the dominant clusters of physical activity and sedentary patterns in Greek preadolescents and to investigate their association with sources of stress. Multivariate data analysis revealed that parental expectations, as a source of stress, were strongly associated with children’s activity-related behaviors. Despite the limitations posed by the study design, our findings are important from a public health perspective. In particular, based on the knowledge that parental expectations, as a stressor in early adolescence, may shape the attitude of children towards physical activity, tailor-made parent–child interventions should be developed for those groups who exhibit the most unhealthy activity-related behavior.

In line with the current literature [9,10], our results revealed that beyond the commonly identified Inactive–Sedentary behavior pattern, different clusters such as Active–Non-screen sedentary and Inactive–Non-Sedentary were also recognized, confirming that multiple clusters of activity-related patterns dominate in early life stages. This finding is justified by the fact that despite the confirmed inverse association between physical activity and sedentary behavior, the effect estimate is small. This has implications that these behaviors do not necessarily replace one another [8] and can even exist simultaneously throughout the day.

In addition, children who followed a sedentary lifestyle pattern were also more likely to be engaged in unhealthy dietary habits with a higher likelihood of consuming fast foods and fewer sleeping hours. These findings are consistent with the literature confirming that obesogenic and unhealthy behaviors are “aggregated” as combined risk patterns [21,22,23], with potential synergistic detrimental effects on well-being [24]. Furthermore, children who were inactive, although non-sedentary, followed less healthy dietary patterns with a higher likelihood of belonging to single-parent families. Indeed, research showed that single-parent families had children who exhibited poorer dietary habits [25] and were prone to sedentariness [26], likely because parents’ involvement in physical activities plays a significant role in children’s physical activity [27].

The present study also found that although the vast majority of children reported stress from various sources (school, family, and friends/peers), a non-significant association was found among the different clusters of activity-related patterns and preadolescent stress. This is in line with the study by Gerber and Puhse (2008) [28], which generally supported the role of physical activity as a moderator of the health–illness relationship. Moreover, a recently conducted meta-analysis found a significant association between mental health and physical activity in adolescents, but not in children [29].

With respect to whether the different causes of children’s stress have the same influence on activity-related behaviors, our findings underlined that only parental expectations showed a significant interaction with children’s physical activity status. In particular, the higher the parental expectations, the higher the sedentary behavior of preadolescents and the lower their physical activity. Although school stress has been recognized as a relatively common stressor in pre-adolescence [14,30], conflict with parents may increase when children ask for greater autonomy as they negotiate their boundaries and responsibilities [31]. Moreover, although meta-analytic findings provide evidence for a positive association between parental educational expectations and a child’s academic achievement [32], when parental expectations exceed the child’s achievement, which puts greater pressure on children’s life, this could cause academic stress via parental pressure among school students [33]. Thus, in the present study, one could assume that parental expectations as a stressor reported by children may imply that their parent’s expectations are greater than their internal ones as regards their academic achievement. It is also worth mentioning that children that followed the “Active-Non-screen sedentary pattern” had the lowest odds of being stressed by their parent’s expectations. This is justified by the fact that all types of sedentary activity do not have the same influence on mental well-being [34]. To this issue, relaxing activities such as playing an instrument may have a beneficial effect on mental well-being [29]. In contrast, children who face stressful events have a higher likelihood of being engaged in screen sedentary activities (i.e., Internet) for fun or socializing in their attempt to manage their bad mood [35]. The literature suggests that culture shapes the interpretation of daily stressful experiences [36]. ‘Albeit parent- and school-related stressors as perceived by adolescents are common among different cultures, differences were observed in coping behaviors” [15]. The significant role of parental expectations as the only stressor on children’s activity-related behavior observed in the sample of Greek pre-adolescents could imply the “grade-hunting” parental attitude, which is common in Greek families Given the bidirectional association between psychological stress and activity-related behaviors [12], the beneficial effects of physical activity on children’s stress levels may be attributed to neurobiological, psychosocial, and behavioral mechanisms [37].

To the best of our knowledge, this is one of the very few studies that have investigated the level of physical activity in pre-adolescence using the concept of clustering various kinds of activities—structured (e.g., sports) or unstructured (e.g., walking)—with sedentary behaviors and sources of stress. However, inherent limitations may exist, principally due to the cross-sectional nature of the study. First, the findings of the present study cannot be generalized to the entire preadolescent population. Additionally, the possible reporting bias of preadolescents cannot be ignored, although the presence of an investigator clarifying any misconceptions about the questionnaire increased the validity of the answers. Moreover, the lack of the use of accelerometers along with the self-reported answers relevant to children’s stress levels could influence the accuracy of the findings.

## 5. Conclusions

Considering the need to promote physical activity in early life stages, the identification of these complex activity-related patterns along with their significant interaction with parental expectations could enhance the effectiveness of targeted behavior change interventions among those parent–child dyads most in need. In particular, tailor-made counselling interventions to parents as regards the influence of their expectations in shaping activity-related behaviors of children are warranted. Moreover, identifying the leading cause of stress is of utmost importance for adopting healthy habits in this vulnerable age group of Greek preadolescents, particularly to fight the burden of childhood obesity. The need for parental education strategies focusing on the crucial role of parents’ expectations as a stressor on their offspring’s activity-related behaviors is highlighted.

## Figures and Tables

**Table 1:** Physical activity and nutritional characteristics of children according to the cluster in which they belong.

	Cluster 1 (Ν = 752)	Cluster 2 (N = 770)	Cluster 3 (Ν = 206)	*p*-Value
**Sleep duration** [hours; Mean (SD)]	9.7 (0.9)	9.7 (1.0)	9.3 (1.1)	**0.002**
**Physical activity characteristics**				
**Way of moving to school** (%)				
*Walking*	49.6	57.2	40.5	**0.013**
*With car*	40	32.9	42.2	
*Other way (e.g., school bus, bicycle)*	10.4	9.9	17.3	
**Studying for school** [Minutes/week; Mean (SD)]	740.7 (342.8)	752.6 (360.0)	857.8 (528.7)	**0.018**
**Computer use** [Minutes/week; Mean (SD)]	406.6 (264.4)	376.6 (241.5)	1007.5 (562.3)	**<0.001**
**Watching TV** [Minutes/week; Mean (SD)]	559.9 (306.7)	549.3 (355.2)	1185.8 (576.1)	**<0.001**
**Reading extracurricular books** [Minutes/week; Mean (SD)]	419.9 (299.8)	549.9 (347.9)	746.7 (619.6)	**<0.001**
**Playing board games** [Minutes/week; Mean (SD)]	390.3 (259.2)	503.3 (342.2)	722.8 (583.9)	**<0.001**
**Playing video games** [Minutes/week; Mean (SD)]	406.6 (278.4)	441.4 (307.1)	1099.1 (619.7)	**<0.001**
**Physical activity** [Minutes/week; Mean (SD)]	184.9 (76.4)	268.8 (128.7)	238.1 (133.8)	**<0.001**
**Screen-based sedentary time** [Minutes/week; Mean (SD)]	1373.2 (573.5)	1367.3 (595.6)	3292.4 (1274.1)	**<0.001**
**Non screen based sedentary time** [Minutes/week; Mean (SD)]	1550.9 (554.5)	1805.8 (715.9)	2327.3 (1365.0)	**<0.001**
**Duration of walking for obligations** (%)				
*<15 min*	43.6	19.6	22	**<0.001**
*15–30 min*	53.8	21.0	28.8	
*31–45 min*	2.6	20.5	24.6	
*46–60 min*	0	16.8	8.5	
*>60 min*	0	22.1	16.1	
**Athlete in extracurricular sports club** (%)				
*No*	3.5	7.5	8.1	**0.018**
*Yes*	96.5	92.5	91.9	
**Frequency of training in extracurricular sports club** (%)				
*1 time/week*	6.4	1.1	3.5	**<0.001**
*2 times/week*	25.2	8.4	13.3	
*3 times/week*	39.3	12.0	34.5	
*4 times/week*	20	15.6	18.6	
*5 times/week*	4.7	31.6	13.3	
*6 times/week*	4.4	31.3	16.8	
**Training duration/time** (%)				
*<* *Approximately 30 min*	5.3	1.6	9.3	**<0.001**
*30–60 min*	40.8	15.0	32.2	
*60–90 min*	42	44.6	43.2	
*>90 min*	11.9	38.8	15.3	
**Years of systematic physical exercise** [Mean (SD)]	4.1 (2.1)	4.8 (2.0)	4.2 (2.1)	**<0.001**
**Nutritional characteristics**				
**Frequency of breakfast consumption** (%)				
*Never/Almost never*	5.6	3.5	6	0.721
*1–2 times/week*	14	10.6	14.5	
*3–4 times/week*	8.9	8.9	12.8	
*5–6 times/week*	7	7.0	6	
*Every day*	64.5	70.0	60.7	
**Number of meals, including snacks** (%)				
*1–2 meals per day*	13	9.6	17.3	0.082
*3 meals per day*	34	34.2	31.8	
*>3 meals per day*	53	56.2	50.9	
**Frequency of fast food consumption** (%)				
*Never/Almost never*	64.6	67.9	40.2	**<0.001**
*1–2 times/week*	33.1	27.9	53.8	
*3–4 times/week*	1.6	2.7	4.3	
*5–7 times/week*	0.7	1.5	1.7	
**Frequency of fast food delivery** (%)				
Never/Almost never	51.3	56.0	40.2	**<0.001**
1–2 times/week	44.7	41.4	53.8	
3–4 times/week	3.1	2.4	4.3	
5–7 times/week	0.9	0.2	1.7	
**KIDMED score** [Mean (SD)]	4.5 (2.2)	5.0 (2.2)	4.7 (2.2)	**0.003**

Notes: SD = Standard Deviation; Cluster 1 was characterized by both physical activity time and sedentary time (both screen-based and non-screen-based), below the average; Cluster 2 was characterized by physical activity and non-screen-based sedentary time above the average, and by screen-based sedentary time below the average; Cluster 3 was characterized by sedentary time (both screen-based and non-screen-based) above the average, and by physical activity below the average; *p*-value was based on the Pearson chi-square test and on the one-way Analysis of Variance (ANOVA) in case of categorical and continuous characteristics, respectively; *p*-values in bold represent the statistically significant differences.

**Table 2:** Demographic, anthropometric, and lifestyle characteristics of the children and their parents, according to the cluster to which they belong.

	Cluster 1 (Ν = 752)	Cluster 2 (Ν = 770)	Cluster 3 (Ν = 206)	*p*-Value
**Children’s demographic characteristics**				
**Age** [in years; Mean (SD)]	11.2 (0.8)	11.2 (0.8)	11.4 (0.8)	0.065
**Sex** (%)				
Boy	40.6	47.7	62.7	**<0.001**
Girl	59.4	52.3	37.3	
**Body Mass Index** [BMI in kg/m^2^; Mean (SD)]	19.2 (3.5)		19.1 (3.6)	0.475
Overweight/Obese (%)	24.5	22.9	33.6	0.129
**Parents’ demographic characteristics**				
**Parents’ age** [in years; Mean (SD)]				
Father	45.9 (5.4)	46.2 (5.2)	46.3 (5.3)	0.564
Mother	41.3 (4.3)	42.0 (4.2)	41.3 (4.6)	0.194
**Marital status** (%)				
Single	13	9.3	4.9	**0.023**
Married	87	90.7	95.1	
**Socioeconomic characteristics**				
**Father’s educational level** (%)				
Basic-secondary	55.8	54.4	68.3	0.150
Higher	44.2	45.6	31.7	
**Mother’s educational level** (%)				
Basic-secondary	49.7	49.0	61.7	0.235
Higher	50.3	51.0	38.3	
**Father’s occupation** (%)				
Unemployed	8.6	5.1	6.7	0.283
Employed	91.4	94.9	93.3	
**Mother’s occupation** (%)				
Unemployed	18.9	15.1	19.6	0.569
Employed	81.1	84.9	80.4	
**Annual family income** (%)				
*<12,000 euros*	24.2	20.4	18.5	0.305
*12,000–18,000 euros*	20.1	24.1	18.5	
*18,000–24,000 euros*	17.3	22.5	27.8	
*24,000–30,000 euros*	19.4	14.2	20.4	
*>30,000 euros*	19	18.8	14.8	
**Parents’ anthropometric and lifestyle characteristics**				
**Father’s physical activity** (%)				
*None*	43.5	39.5	39.3	0.804
*1–2 times/week*	35.2	36.7	41.1	
*>3 times/week*	21.3	23.8	19.6	
**Mother’s physical activity** (%)				
*None*	37.4	37.0	38.9	0.503
*1–2 times/week*	41	37.0	33.3	
*>3 times/week*	21.6	26.0	27.8	
**Body Mass Index** [BMI in kg/m^2^; Mean (SD)]				
*Father*	26.8 (3.4)	27.2 (3.6)	26.4 (3.8)	0.326
*Overweight/Obese (%)*	67.7	70.5	62.3	0.581
*Mother*	23.8 (3.8)	24.1 (4.2)	23.4 (3.7)	0.563
*Overweight/Obese (%)*	29.5	30.1	26.9	0.948

Notes: SD = Standard Deviation; Cluster 1 was characterized by both physical activity time and sedentary time (both screen-based and non-screen-based) below the average; Cluster 2 was characterized by physical activity and non-screen-based sedentary time above the average and by screen-based sedentary time below the average; Cluster 3 was characterized by sedentary time (both screen-based and non-screen-based) above the average, and by physical activity below the average; *p*-value was based on the Pearson chi-square test and on the one-way Analysis of Variance (ANOVA) in the case of categorical and continuous characteristics, respectively; *p*-values in bold represent the statistically significant differences.

## Data Availability

The data presented in this study are available upon request from the corresponding author. The data are not publicly available due to privacy and ethical reasons.
